# Re-Tooling of γδ T Cells for Cancer Immunotherapy Using Advanced Manufacturing and Genetic Engineering

**DOI:** 10.3390/cells15060494

**Published:** 2026-03-10

**Authors:** Benjamin J. L. Lim, John Maher

**Affiliations:** 1 University of Edinburgh Medical School, University of Edinburgh, Little France Crescent, Edinburgh EH16 4UU, UK; s2140759@ed.ac.uk; 2 Department of Precision and Population Oncology, Guy’s Hospital, King’s College London, London SE1 9RT, UK; 3 Leucid Bio Ltd., Guy’s Hospital, Great Maze Pond, London SE1 9RT, UK; 4 Department of Immunology, Eastbourne Hospital, Kings Drive, Eastbourne BN21 2UD, UK

**Keywords:** immunotherapy, malignancy, γδ T cell, chimeric antigen receptor

## Abstract

**Highlights:**

**What are the main findings?**

- Vδ2 cells are the predominant circulatory subtype.
- Vδ1 cells are highly tissue tropic.

**What are the implications of the main findings?**

- Allogeneic γδ T cells are under development for cancer immunotherapy
- Innovative genetic engineering and manufacturing strategies may be used to boost potency.

**Abstract:**

Adoptive immunotherapy using ex-vivo-amplified autologous αβ T cells has achieved notable success in the treatment of diverse cancer types. Pre-eminent among these developments has been the advent of chimeric antigen receptor (CAR) T cell therapy, which has revolutionised the treatment of selected haematological malignancies. However, autologous CAR T cell immunotherapy is poorly scalable and has demonstrated limited efficacy against solid tumours. Accordingly, there has been significant interest in alternative strategies that may bridge these gaps. The use of γδ T cells is an attractive alternative since they possess intrinsic anti-tumour activity and do not elicit graft versus host disease (GvHD) when employed as an allogeneic drug product. In this review, we evaluate the potential use of γδ T cells for cancer immunotherapy and how manufacturing and genetic engineering refinements can be used to potentiate this activity. We also summarise current clinical experience with CAR γδ T cell therapies and discuss the implications of these findings for the next generation of cellular immunotherapies.

## 1. Introduction

T lymphocytes are haematopoietic cells of the lymphoid lineage and play a key role in orchestrating immune responses against pathogenic insults, including cancer. To achieve this, T cells recognise aberrant attributes of diseased cells via a clonotypic T cell receptor (TCR), leading to a coordinated immune response. Approximately 95% of circulating T cells express an αβ TCR, which generally recognise processed peptide antigens in a major histocompatibility complex (MHC)-dependent manner. In recent decades, immunotherapies that re-invigorate or re-program αβ T cells have become a cornerstone of cancer treatment.

One rapidly advancing form of cellular immunotherapy involves the genetic modification of αβ T cells to express a synthetic receptor known as a chimeric antigen receptor (CAR) [1]. Chimeric antigen receptors are recombinant fusion proteins with a highly modular architecture. Antigen recognition is most commonly achieved using a single chain variable fragment (scFv), formed by linking the variable domains of antibody heavy and light chains with a short linker protein. This is generally followed by a hinge or spacer region, a transmembrane domain, and a bespoke intracellular signalling endodomain. Importantly, CARs permit the engineered T cells to engage native cell surface antigens in an MHC-independent manner, broadening applicability across different patient populations and circumventing immune evasion through MHC class I downregulation. Upon antigen engagement, CAR T cells typically mediate tumour cell lysis and undergo a burst of proliferation and cytokine release. The greatest clinical success has been achieved using second-generation CARs, originally conceived by Finney et al. [2,3], which incorporate a fused co-stimulatory unit (such as CD28 or 4-1BB) and activating module, most commonly derived from CD3ζ. Using this platform, remarkable clinical efficacy has been observed in selected B cell and plasma cell malignancies, targeting the lineage-associated cell surface markers CD19 and B cell maturation antigen, respectively [4]. However, the scalability of manufacture of autologous products is challenging and fewer than 20% of eligible patients currently receive approved CAR T cell products (https://www.iqvia.com/locations/united-states/library/white-papers/the-path-to-car-t-cell-therapy, accessed on 21 January 2026). Moreover, donor-to-donor variability and pronged vein-to-vein times further hinder clinical applicability.

An additional key limitation of CAR T cell immunotherapy is sub-optimal efficacy against solid tumours. Target selection is the first key challenge; solid tumour antigens generally lack specificity for malignant cells and are expressed heterogeneously within primary and metastatic tumours [5]. Reflecting this, well over 100 solid tumour targets have been evaluated in pre-clinical CAR T cell studies [6]. Efficient trafficking and infiltration of CAR T cells also poses a major obstacle [7], particularly given the high interstitial pressure within solid tumours. The tumour microenvironment (TME) is the ultimate battleground for these cells but imposes physical, biological and chemical barriers to success. It is typically characterised by hypoxia, oxidative stress, nutrient deprivation, metabolic dysfunction and acidosis, all of which impair T cell function [8,9]. In addition, the TME contains profoundly immunosuppressive regulatory T cells (Tregs), myeloid-derived suppressor cells (MDSCs), macrophages and immunosuppressive mesenchymal stromal cells that dampen anti-tumour immunity [10,11]. These effects are compounded by inhibitory soluble mediators such as transforming growth factor (TGF)-β, adenosine, indoleamine dioxygenase and tryptophan dioxygenase [12], as well as tumour-intrinsic upregulation of immune checkpoint ligands such as programmed death ligand 1 (PD-L1) [10,13]. Collectively, these factors drive CAR T cell dysfunction, exhaustion and loss of durable anti-tumour effect [14,15]. Consequently, no CAR T cell product has received regulatory approval for solid tumour treatment.

Given the challenges surrounding the manufacture of autologous αβ CAR T cells and their limited success against solid tumours, there has been increasing interest in exploring an alternative cellular chassis for CAR engineering [8]. γδ T cells constitute approximately 1–10% of circulating lymphocytes and possess potent intrinsic anti-tumour effector functions [16]. Notably, across 39 cancer types, the presence of tumour-infiltrating γδ T cells has been identified as one of the strongest immune correlates of favourable prognosis [17]. In contrast to their αβ counterparts, γδ T cells express a more restricted TCR repertoire derived from seven Vγ chains (Vγ2, 3, 4, 5, 8, 9 and 11) and four Vδ chains (Vδ1, 2, 3 and 5) [18]. They recognise metabolic, signalling, stress-associated and genomic attributes of diseased cells in an MHC-independent manner via the γδ TCR itself and a range of stress or natural killer cell (NK) receptors, including NKG2D, DNAX accessory molecule-1 (DNAM-1), NKp30, NKp44 and NKp46 [19,20,21,22]. Accordingly, they are considered a bridge between innate and adaptive immunity, and have been described as ‘nature’s CAR T therapy’ [23].

Activated γδ T cells exert multiple anti-tumour effector mechanisms. These include the secretion of interferon γ (IFN-γ) and tumour necrosis factor α (TNF-α), which enhance antigen presentation, inhibit angiogenesis and promote dendritic cell (DC) maturation [19,20]. γδ T cells can directly promote tumour cell death via the perforin–granzyme pathway, FAS–FAS ligand interactions and TNF-related apoptosis-inducing ligand (TRAIL) [24]. CD16-expressing γδ T cells can elicit antibody-dependent cellular cytotoxicity (ADCC). γδ T cells have also been reported to possess antigen presentation capacity [25], including the capacity to undertake cross presentation [26], even when engineered to express a CAR [27]. However, γδ T cell function within the TME is inhibited by hypoxia in a manner that can be alleviated by inhibition of HIF (hypoxia-inducible factor)-1 signalling [28]. Although γδ T cells have demonstrated encouraging pre-clinical and early clinical activity, there are currently no FDA-approved γδ T cell-based or CAR γδ T cell therapies. Moreover, like many other elements of the immune system, γδ T cells can exert double-edged effects with respects to cancer. For example, some γδ T cell subsets produce interleukin (IL)-17 [29], while others generate amphiregulin [30], both of which have tumour-promoting properties.

In this review, we evaluate the prospects of γδ T cells as cell immunotherapy effectors, with particular emphasis on expansion methods and engineering strategies designed to overcome the challenges posed by solid tumours.

## 2. γδ T Cell Subsets

**Figure 1:**
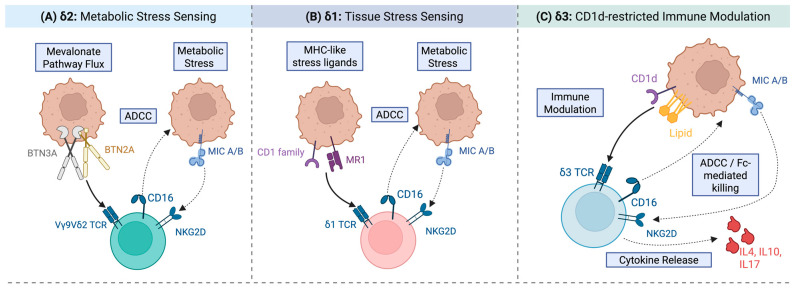
The three main γδ T cell subtypes are characterised by the expression of a Vδ2, Vδ1 or Vδ3 TCR subunit. Vδ2 γδ T cells are activated by phosphoantigens, which are products of the mevalonate pathway. Mevalonate pathway flux is increased in response to metabolic stress within the tumour microenvironment, which engages the Vδ2 TCR via the BTN2A1-BTN3A1-BTN3A2 complex. In addition, NKG2D engagement by stress-induced ligands such as MICA/B provides a co-stimulatory signal. Across all γδ T cell subtypes, expression of CD16 (FcγRIII) enables ADCC against opsonised tumour cells. The Vδ1 T cell receptor recognises stress-associated ligands presented by MHC-like molecules, including members of the CD1 family and MR1. Vδ1 T cells similarly express CD16 and NKG2D. In this subtype, the integration of innate stress signals and TCR-dependent recognition enables broad tumour surveillance. Vδ3 T cells also recognise lipid antigens through CD1d-restricted immune sensing and exhibit prominent immunomodulatory functions through cytokine secretion (e.g., IL-4, IL-10, IL-17). In doing so, this subset bridges cytotoxic effector activity with regulatory and inflammatory immune responses.

In humans, there are three principal γδ T cell subtypes that are characterised by the expression of either a Vδ1, Vδ2 or Vδ3 TCR subunit [19,31] (Figure 1). A key distinguishing feature between these subtypes is their propensity for distribution across different tissues. The main population found in the bloodstream is the Vδ2 subtype, in which the partner TCR subunit is almost invariably Vγ9. γδ2 cells account for approximately 1–5% of all T cells in adult peripheral blood mononuclear cells (PBMC), of which up to 95% are of the δ2 subtype [32]. Uniquely, they are activated by phosphoantigen (pAg) intermediates of the mevalonate pathway [33,34,35]. While many of the most potent pAgs are generated by microbes, some human mevalonate pathway intermediates also act as pAgs. Detection of pAgs within human target cells is mediated by the BTN2A1-BTN3A1-BTN3A2 butyrophilin family complex, most likely by an “inside out” signalling model [36]. Binding of pAgs to the intracellular B30.2 domain of BTN3A1 alters the conformation of the butyrophilin complex, enabling it to bind the γδ TCR [37,38]. As flux through the mevalonate pathway is increased in malignant cells, Vδ2 cells serve as an intrinsic surveillance system for a key hallmark of transformation [39]. Moreover, pAg levels can be artificially elevated using aminobisphosphonate drugs (N-BPs), which inhibit the rate-limiting enzyme of the mevalonate pathway, farnesyl pyrophosphate synthase [40]. Aminobisphosphonates have high affinity for hydroxyapatite in bones and teeth but are poorly tropic for extraosseous tumours. However, this can be enhanced using nanoparticle delivery systems, potentiating tumour sensitivity to Vδ2 cells [41]. Butyrophilins are also upregulated in an AMP-activated protein kinase (AMPK)-dependent manner during metabolic stress [42]. This provides another potential approach to enhance tumour cell recognition by these cells using AMPK agonists such as metformin [43]. Vδ2 T cells can also recognise a complex formed by F1-ATPase in conjunction with ApoAI and MutS homolog 2 [44], in addition to some proteins that redistribute to the cell surface under conditions of cell stress [45].

In contrast to the mainly circulatory nature of Vδ2 cells, Vδ1 T cells are primarily found in skin, mucosal or subcutaneous tissues, the intestinal epithelium, the lungs, reproductive organs, the liver and the spleen [19]. Within tissues, Vδ1 cells receive tropic signals from cognate butyrophilins, an interaction that tumours may corrupt in an effort to exclude these cells from the TME [46]. Nonetheless, the presence of tumour-infiltrating Vδ1 but not Vδ2 cells has been linked to improved prognosis in some solid tumour types [46,47,48]. Moreover, they represent the primary form of tumour-infiltrating lymphocytes with anti-tumour activity in some cancers, such as paediatric neuroblastoma [49]. Vδ1 T cells have also been reported to be less prone to activation-induced cell death (AICD) in contrast to their Vδ2 cell counterparts [50]. Although ligands for the Vδ1 TCR are not fully characterised, some demonstrate restriction to members of the CD1 family or MR1 [51]. These are also targets of other unconventional T cell types including NKT (natural killer T) cells and MAIT (mucosal-associated invariant T) cells, highlighting some overlap in antigen recognition mechanisms that promote the activation of these distinct cell types. Indeed, evidence has recently been presented that these cells may compete within a homeostatic niche, enabling them to deliver complementary and redundant functionality [52]. Vδ1 T cells also recognise annexin A2 [53] and ephrin type A receptor 2 [54], both of which are stress-induced molecules. Additionally, these cells have been linked to the recognition of proteins that undergo ectopic cell surface expression under conditions of cell stress, including nucleolin [45].

Vδ3 cells are rare in the peripheral circulation and localise predominantly in the gastrointestinal tract and liver [55]. They engage similar ligands to Vδ1 cells, and similar to other subtypes they also express CD16 to enable them to elicit ADCC. Vδ3 cells also respond to cells infected with cytomegalovirus [55]. Moreover, Vδ1 and Vδ3 T cells express high levels of NK receptors and have been implicated as effectors of immune checkpoint blockade in solid tumours that lack β2 microglobulin (and by inference HLA class I) [56].

## 3. Expansion Methods for γδ T Cells

### 3.1. Expansion of Vδ2 T Cells Using Synthetic Phosphoantigens and Aminobisphosphonates

As indicated above, pAgs are naturally occurring intermediates of mevalonate pathway metabolism. Consequently, expansion of PBMC-derived Vδ2 cells for clinical studies has relied heavily on the use of synthetic pAgs such as bromohydrin pyrophosphate (BrHPP), also known as phosphostim [57,58]. Zoledronic acid (ZOL) is an N-BP that has also been widely used for the expansion of Vδ2 cells in conjunction with IL-2, achieving purities in excess of 90% (for detailed protocols see [57,59]). Moreover, this approach has also been shown to be effective using Vδ2 cells from cancer patients [60] and is compatible with both transient [61,62] and stable CAR engineering [63]. A GMP-compatible method to expand ZOL-stimulated Vδ2 cells has recently been described [64]. Vitamin C supplementation has also been shown to boost cell cycle progression, expansion and effector function of ZOL/pAg-stimulated Vδ2 cells [65]. It should be noted, however, that the quality of expanded cells across these various protocols has exhibited considerable donor-to-donor variability [66]. Accordingly, many groups have set out to further optimise Vδ2 cell expansion with the addition of cytokines including IL-2 + IL-4 [67], IL-15 [68], IL-18 [69] or IL-2 + IL-12 + CD137 ligand [70]. In the latter case, the N-BP alendronic acid proved more effective than ZOL as an activating stimulus.

A key limitation of methods that rely on ZOL and other pAgs is the induction of exhaustion and AICD of Vδ2 cells [71]. Furthermore, sustained mevalonate pathway inhibition by drugs such as ZOL impairs Vδ2 T cell function, accompanied by upregulation of the TIM3 immune checkpoint [72].

### 3.2. Expansion of Vδ2 Cells Using Butyrophilin Agonists

Given the established role of the butyrophilin family complex in the recognition of pAgs, BTN3A1 agonistic monoclonal antibodies have been developed that induce conformational changes that drive Vδ2 T cell expansion and effector function [73]. Studies in vitro and in murine haematological cancer models have demonstrated that such antibodies enhance the anti-tumour activity of Vδ2 T cells [73,74]. BTN3A1 agonists have also been engineered as bispecific antibodies to promote tumour localisation, raising the possibility of their use as adjuncts to CAR-engineered Vδ2 cell therapies [75,76]. The clinical utility and scalability of butyrophilin agonists for manufacturing platforms remain to be fully defined.

### 3.3. Expansion of Vδ2 Cells in the Presence of Transforming Growth Factor β

Transforming growth factor β (TGF-β) is a pleiotropic cytokine that has classically been associated with pro-tumour and immunosuppressive activities. When added to Vδ2 cells, TGF-β induces some regulatory properties in these cells, including FoxP3 expression [77,78]. Intriguingly, however, the Kabelitz group showed that Vδ2 cells expanded in the presence of TGF-β also produce high levels of IL-9, a cytokine linked to anti-tumour activity [79]. Subsequently, the same group reported that TGF-β enhanced the cytolytic activity of these cells [80] and that full induction of regulatory activity by this cytokine additionally required epigenetic modification using vitamin C [78]. Beatson and Parente-Pereira et al. demonstrated that expansion of either ZOL or pan γδ TCR antibody-stimulated γδ T cells in the presence of TGF-β and IL-2 resulted in enhanced efficacy in models of both solid and haematological malignancy [81,82]. These so-called “T2 cells” exhibited a distinctive phenotype similar to that reported by the Kabelitz group, with elevated expression of tissue residency markers (e.g., CD103) and the bone marrow homing chemokine receptor, CXCR4. Superior efficacy of these cells was also confirmed when engineered to express a tumour-specific CAR. Importantly, T2 cells demonstrated resistance to immunosuppressive actions of prostaglandin E2. They were also immune to suppressive actions of TGF-β itself, which otherwise reduced tumour cell killing and degranulation when added to ex-vivo-expanded Vδ2 cells for 72 h [83]. A similar beneficial effect of TGF-β on ZOL-expanded Vδ2 cells was shown in models of osteosarcoma [84].

### 3.4. Vδ1 T Cell Expansion

Historically, efforts to expand Vδ1 T cells have used mitogenic plant lectins such as concanavalin A and phytohemagglutinin, which do not have clinical translatability [85]. To address this limitation, Almeida et al. described a clinical grade expansion protocol for Vδ1 cells (referred to as delta 1 T or “DOT cells”) [86]. The protocol involved a two-step magnetic bead sorting process followed by 1 week of culture in IL-21, IL-1β, IL-4, interferon (IFN)-γ and the anti-CD3 monoclonal antibody clone OKT3, and a second week of culture in IL-15, IFN-γ and OKT3. The expanded DOT population exhibited high expression of NK-cell-associated cytotoxic receptors including NKG2D, NKp30, NKp44, DNAM-1 and 2B4. The efficacy of CD123 CAR-targeted DOT cells in patient-derived xenograft models of acute myeloid leukaemia has been demonstrated [87].

To build on this, Ferry et al. designed a one-step procedure to expand DOTs from αβ T-cell- and CD56-depleted PBMCs [88]. The authors did not make a firm recommendation on the need for additional depletion of Vδ2 T cells from these cultures, given the lack of commercially available GMP reagents that could be used to achieve this. The protocol could be performed on cryopreserved PBMCs and was compatible with retroviral CAR transduction. Furthermore, DOT cells engineered to express B7-H3-specific CARs were shown to exhibit high cytotoxicity against a range of tumour targets.

In the commercial sector, Adicet Bio has developed a proprietary Vδ1 antibody-based activation strategy followed by CAR transduction and IL-2-driven large scale expansion of these cells from donor leukapheresis material [89]. Final cell products are immunomagnetically depleted of αβ cells using the CliniMACS^®^ system (Miltenyi Biotec, Sydney, Australia), which is fully compliant with clinical manufacturing requirements.

### 3.5. Expansion of γδ T Cells Using Artificial Antigen-Presenting Cells

Multiple studies have described the use of artificial antigen-presenting cells (aAPCs) as an alternative approach to achieve high-efficiency expansion of γδ T cells. Most commonly, feeder cells have been derived from the K562 cell line due to its lack of MHC I and II expression. Illustrating this, Deninger et al. first depleted CD56^+^ (mainly NK) cells, after which γδ T cells were subjected to positive selection. Cells were serially stimulated with irradiated K562 feeder cells that co-expressed 4-1BBL, CD86 and membrane-bound (mb)IL-15, together with exogenous IL-2 and IL-21 [90]. A highly pure culture of γδ T cells containing both Vδ1 and Vδ2 subtypes emerged after 22 days. Vδ2 cells have also been expanded using K562 cells engineered to co-express CD80, 4-1BB ligand and CD83 [91] or using the G-Rex^®^ platform (Wilson Wolf, New Brighton, MN, USA, gas permeable rapid expansion, see below) supplemented with OKT3 and K562 feeder cells that co-express CD64, CD86 and CD137 ligand [92].

CAR-expressing γδ T cells may also be expanded using related aAPC systems. Illustrating this, CD19 CAR-engineered γδ T cells were purified using paramagnetic beads and selectively expanded in soluble IL-2 and IL-21 by repeated stimulation with irradiated K562 cells that expressed CD19, CD64, CD86, CD137 ligand and mbIL-15 [93]. K562 feeder cells engineered to express mbIL-21 have been used to expand γδ T cells, leading to preferential enrichment of Vδ1 cells [94]. The compatibility of the system with CAR engineering was also demonstrated.

Highly pure Vδ2 T cell cultures have been expanded following positive selection using antibody-based magnetic cell separation (MACS^®^) followed by stimulation in the presence of autologous irradiated feeder PBMCs with BrHPP and IL-2 [95]. Similarly, irradiated PBMCs and Epstein–Barr virus-transformed B lymphoblastoid feeder cells have been used for this purpose [96].

Jiang et al. described a large scale manufacturing process for CAR-engineered Vδ1 T cells, employing irradiated feeder cells [97]. Cells were enriched and activated by immunomagnetic positive selection using an anti-TCR Vδ1 antibody. The CAR was co-expressed with IL-2 by lentiviral transduction and cells expanded thereafter in a Xuri^TM^/WAVE bioreactor^TM^ with IL-2, IL-15 and IL-21 together with K562 aAPCs that expressed CD64, CD86, CD137 ligand and IL-15.

Despite the efficient expansion and functional potency of cell products generated using these methods, it should be noted that the use of genetically modified feeder cell lines introduces additional manufacturing complexity, regulatory burden and safety considerations.

### 3.6. Antibody-Based Expansion of γδ T Cells

There have been several reports in which expansion of γδ T cells has been triggered using immobilised Vδ1 or Vδ2 antibodies. Illustrating this, Bridge et al. [98] have recently described a feeder-cell-free method to expand either pan γδ T cells, as well as Vδ1 or Vδ2 subsets, using cognate antibodies plus soluble anti-CD28. Cells were also amenable to non-viral genetic modification to achieve CAR expression alone, or together with base editing to inactivate Fas, PD1 and CISH (cytokine-inducible SH2 containing protein), thereby reducing AICD and enhancing anti-tumour activity.

### 3.7. Genetic Engineering of γδ T Cells

In many reports, similar methods used to transduce αβ T cells have also been used for γδ T cells. However, protocols and reagents may require specific optimisation for γδ T cells. Accordingly, lentiviral vectors pseudotyped with a vesicular stomatitis virus envelope proved sub-optimal for transduction of Vδ2 T cells, in contrast to αβ T cells [99]. By contrast, CAR engineering using a baboon endogenous retrovirus envelope resulted in enhanced gene transfer efficiency and anti-tumour activity of these cells [99]. Vitamin C has also been shown to enhance the viability of Vδ2 T cells following lentiviral transduction [100].

Other groups have emphasised the desirability of generating engineered products in which multiple γδ T cell subtypes are represented. Illustrating this, Snyder et al. described a system whereby cells were activated using a pan γδ TCR antibody and then expanded using irradiated K562 feeder cells that co-express mbIL-21 and 4-1BB ligand [101]. Site-specific delivery of a CD38-specific CAR was achieved using CRISPR/AAV-mediated knock in at the CD38 locus, thereby overcoming fratricide of the cells and maintaining anti-tumour activity.

### 3.8. Scalable and Automated Manufacturing Platforms for CAR γδ T Cells

To date, many γδ T cell expansion and engineering approaches have been developed primarily in small-batch, laboratory-scale settings aimed at biological discovery rather than large-scale manufacture. Such techniques are vulnerable to batch variability, operator dependence, contamination and limited scalability [102]. As T-cell-based therapies progress towards allogeneic and off-the-shelf clinical use, manufacturing scalability, reproducibility and production costs become key variables for clinical translation. Upscaled manufacturing platforms that reduce reliance on manual and open culture workflows are well defined for CAR αβ T cell therapies but require further definition for γδ T cell workflows.

Ideally, end-to-end systems are fully closed and automated manufacturing platforms that integrate cell selection, activation, genetic modification, expansion, washing and formulation within a single GMP-compliant workflow. Platforms such as the Miltenyi Biotec CliniMACS Prodigy^®^, which are well established for αβ CAR T cell manufacture, have been applied to γδ T cell processing [103]. Apheresis material from healthy donors was activated with repeated addition of ZOL and IL-2 followed by depletion of αβ T cells.

The Lonza Cocoon^®^ (Lonza, Basel, Switzerland) employs a parallelised, cassette-based manufacturing architecture and has been developed to support scalable and automated CAR T cell manufacture, minimising user interactions [104]. As such, it may be especially well suited to banked manufacturing models anticipated for off-the-shelf γδ T cell therapies. Although γδ-specific Cocoon^®^ workflows have not yet been formally described, the platform’s design features closely align with the manufacturing requirements of allogeneic CAR γδ T cell products.

G-Rex^®^ culture vessels have also been used by a number of groups to achieve γδ T cell expansion, including CAR-engineered cells [90,92,105]. Cells settle to the bottom of the culture vessel where they form a monolayer in immediate proximity to the gas-permeable membrane. Accordingly, the system supports high-density cell growth through enhanced oxygen diffusion and reduced media handling. Moreover, the system is compatible with the use of engineered irradiated K562 feeder cells to stimulate γδ T cells [92].

Additional closed or semi-automated platforms developed for CAR T cell manufacturing may further support scalable CAR γδ T cell production. These include automated end-to-end systems such as Cytiva Sefia™, bag-based rocking bioreactors such as Xuri™ (Cytiva, Marlborough, MA, USA) and closed downstream processing technologies including CT Rotea™ and kSep^®^, which enable automated washing, concentration and formulation. While γδ T-cell-specific implementations of these systems remain limited, they represent enabling infrastructure that can be combined with tailored activation and expansion strategies to support reproducible manufacture at scale.

### 3.9. Donor Selection

As indicated above, ex vivo expansion of γδ T cells is subject to considerable donor-to-donor variability. Consequently, in an effort to enhance the standardisation of cell banks derived from allogeneic healthy donors, there has been interest in the identification of biomarkers that predict improved expansion and fitness of these cells. CD16 expression on fresh Vδ2 cells has been identified as one such candidate, correlating with enhanced functionality of ex-vivo-expanded cells [106]. CD16 has also been linked to enhanced cytotoxic activity of Vδ2 cells [107,108]. Elevated expression of TIGIT on ex-vivo-expanded Vδ2 cells has also been linked with enhanced activation, cytokine release and tumour cell killing activity [109]. However, Bold et al. reported that degranulation of ex-vivo-expanded Vδ2 cells correlated negatively with baseline CD16 expression and positively with activation marker levels [110]. Moreover, expression of CXCR3 on unstimulated cells correlated with ADCC activity of expanded cells [110].

## 4. Novel Genetic Engineering Strategies for γδ T Cells

### 4.1. CAR Signalling Architecture for γδ T Cell Therapy

Studies that simultaneously evaluate the function of CAR-engineered αβ and CAR γδ T cells are limited. Li et al. performed exactly this comparison for a CD28-containing second-generation CAR targeted against prostate stem cell antigen (PSCA), comparing αβ, Vδ1 and Vδ2 cells [111]. All three performed equivalently in xenograft models of pancreatic cancer, although γδ T cells resulted in less cytokine release syndrome and GvHD.

**Figure 2:**
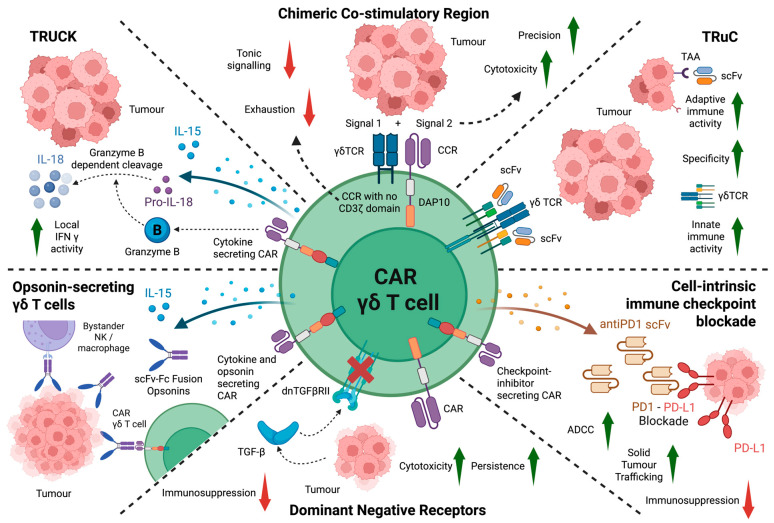
Overview of engineering strategies to optimise γδ T cell-centric anti-tumour function. γδ CARs can be armoured to secrete cytokines, opsonins and immune checkpoint inhibitors, improving localisation of effector function within the TME. T cells redirected for universal cytokine killing (TRUCKs) for γδ T cell immunotherapy may be designed to secrete IL-15 and IL-18 to enhance γδ T cell anti-tumour effects. Local cytokine release upon tumour engagement may limit systemic toxicity while amplifying γδ T cell and bystander immune responses. Similarly, opsonin-secreting γδ T cells coat tumour cells with scFv–Fc fusion opsonins, recruiting bystander NK cells and macrophages and promoting Fc-mediated effector functions, including ADCC. A combined cytokine and opsonin secretion strategy may further enhance local immune activation and tumour clearance. Checkpoint-inhibitor-secreting constructs improve CAR γδ T cell persistence within TMEs that exert immunosuppressive effects through immune checkpoints. CAR γδ T cells have been designed to secrete anti-PD-1 scFv, enabling local PD-1–PD-L1 blockade at the tumour site to enhance ADCC, tumour trafficking and effector function. In a similar manner, expression of dominant-negative TGF-β receptor II (dnTGFβRII) renders CAR γδ T cells resistant to TGF-β-mediated immunosuppression, improving cytotoxicity and persistence within immunosuppressive TMEs. CAR alternatives have been explored to reflect and utilise γδ T cell effector functions more closely. Chimeric co-stimulatory receptors (CCR) lack activating modules such as CD3ζ, reducing ligand-independent tonic signalling, which has been associated with γδ T cell exhaustion. These γδ T cells can be engineered with dual signalling inputs, combining the endogenous γδ TCR (signal 1) and a CCR (signal 2) to localise responses and potentiate cytotoxic function. Finally, TCR fusion constructs (TRuCs) directly fuse an scFv to CD3ε, preserving physiological TCR signalling architecture. This design allows antigen specificity for adaptive immune activation while retaining γδ T cell innate immune function. These γδ T cell-centric CAR strategies are modular and may be explored in combination, and with novel designs, to collectively enhance tumour specificity, cytotoxicity, persistence and immune modulation.

Existing CAR designs have been optimized almost exclusively for αβ T cells, which rely on CD3ζ-based activation domains to drive activity. Unlike αβ T cells, γδ T cells also integrate distinct signals from stress ligands and both metabolic and genomic stress. Recent conceptual frameworks, therefore, propose a γδ T cell-centric engineering approach, in which CARs are designed to cooperate with γδ T cell innate sensing and signalling networks [112]. These approaches are summarised in Figure 2.

### 4.2. Novel γδ T Cell CAR Architectures

Many CARs containing CD3ζ signalling domains can induce ligand-independent (“tonic”) signalling of αβ T cells, resulting in chronic activation, premature differentiation and functional exhaustion [113]. Importantly, this phenomenon has also been reported in CAR-engineered γδ T cells, associated with increased exhaustion marker expression [114].

Fisher et al. undertook a comparative analysis in Vδ2 cells between a typical CD3ζ-containing conventional CAR and a chimeric co-stimulatory domain receptor (CCR) that contained the DAP10 co-stimulatory motif but no activating domain [115]. Using this approach, they created a Boolean logic gate in which the γδ itself TCR provided a tumour-dependent activation signal while the CCR provided a tumour-responsive amplifying co-stimulatory signal [114]. A notable advantage of this arrangement was the abrogation of tonic signalling, while preserving selective anti-tumour activity. This suggests that alternative CAR signalling architectures that decouple antigen recognition from CD3ζ activation may be better suited to enhance CAR γδ T cell function.

IN8Bio demonstrated a proof-of-concept model for the utility of a non-signalling CAR in Vδ2 cells, in which the CD3ζ domain was removed from a traditional second-generation CD19-specific CAR. The non-signalling CAR enabled the selective destruction of malignant but not healthy B cells by Vδ2 cells [116]. However, killing of K562 target cells that lacked CD19 was equivalent whether the non-signalling CAR was present or absent, confirming that the ability of the non-signalling CAR to co-localise the Vδ2 cells with malignant B cells enabled their selective elimination.

### 4.3. Cytokine Armouring of γδ T Cells

Armoured CARs, also known as TRUCKs (T cells redirected for universal cytokine-mediated killing) are fourth-generation CAR systems in which the T cells are engineered to secrete a cytokine [117]. As indicated above, several cytokines have been used to facilitate the manufacture of γδ T cell products, supporting the hypothesis to integrate genetic engineering approaches for intrinsic cytokine release by these cells.

Interleukin 15 is a key stimulator of γδ T cell expansion and effector function [68]. Mesothelin CAR Vδ2 cells demonstrated enhanced tumour control when armoured to produce IL-15 [106]. Adicet Bio’s ADI-002 product consists of allogeneic Vδ1 cells engineered to co-express a glypican 3-specific CAR and IL-15, leading to enhanced efficacy in pre-clinical xenograft models of hepatocellular carcinoma [118]. IN8Bio’s non-signalling CAR technology described above has also been paired with IL-15 production by engineered Vδ2 cells. Once again the selective targeting of malignant but not healthy cells was demonstrated using CARs targeting CD19, CD33 and CD123 [119]. Similarly, opsonin-engineered γδ T cells (see below) have been armoured to secrete IL-15 in combination with IL-15 receptor α [120].

Interleukin 18 exerts several pro-inflammatory effects including the enhancement of T cell cytolytic activity, proliferation and IFN-γ release [121]. However, increased circulating levels of IL-18 and its key mediator, IFN-γ, have also been linked to an increase in toxicities such as cytokine release syndrome [121]. To mitigate this risk, a range of strategies have been employed, most notably the expression of IL-18 under the transcriptional control of a nuclear factor of activated T cell (NFAT)-based promoter [122]. In the context of γδ T cells, Hull et al. developed an alternative strategy whereby the proteolytic cleavage site in the latent precursor of IL-18 (pro-IL-18) was modified to one that is optimally processed by granzyme B [123]. Through these means, the release of biologically active IL-18 is restricted to circumstances whereby the host γδ T cell is activated. Using this approach, the anti-tumour activity of CAR engineered Vδ2 cells was markedly potentiated. Mechanistically, this was accompanied by upregulated mitochondrial mass and expression of the GLUT1 glucose transporter and CD98 amino acid transporter. Notably, these effects of IL-18 were unique to Vδ2 γδ but not αβ T cells. Moreover, recent data indicate that IL-18 also potentiates the anti-tumour activity of Vδ1 cells [124].

### 4.4. Opsonin-Secreting γδ T Cells

As indicated above, traditional CARs have been linked to undesirable tonic signalling when expressed in γδ T cells. Fowler et al. have described an alternative strategy whereby cells were engineered to secrete tumour-specific scFv–Fc fusions that served as a bridge between tumour cells and γδ T cells, in addition to bystander Fc-expressing cell types [120]. To improve persistence, cells co-expressed IL-15 in combination with IL-15 receptor α, enabling improved control of an orthotopic osteosarcoma xenograft when compared to CAR αβ T cells.

### 4.5. Dominant Negative Receptors

Tissue-resident (hepatic) γδ T cells are suppressed in a TGF-β-dependent manner [125]. Accordingly, investigators developing Vδ1 T cell-based therapeutics have also sought to modulate reactivity to this cytokine. Adicet Bio’s ADI-270 consists of a CD27-targeted anti-CD70 CAR Vδ1 T cell product that incorporates a dominant-negative TGF-β receptor II (dnTGFβRII) [126]. A preclinical evaluation demonstrated potent in vitro cytotoxicity, resistance to immunosuppressive effects of TGF-β and a favorable cytokine profile compared to αβ CAR counterparts. The anti-tumour response was confirmed in multiple xenograft models. Moreover, alloreactive host cell rejection was mitigated by its CD70 targeting mechanism, which identifies transiently activated T and NK cells.

### 4.6. Cell-Intrinsic Immune Checkpoint Blockade

Solid tumours may engage inhibitory immune checkpoints via ligands such as PD-L1. Such targets have become the focus of immune checkpoint inhibitor therapies for a number of cancers [127]. It has also been shown that PD1 expression impairs ADCC by Vδ2 T cells against follicular lymphoma in a manner that was alleviated by PD1 blockade [128]. Targeting this, Zhang et al. developed a CAR γδ T cell therapy that secretes an anti-PD1 scFv, neutralising the inhibitory immune signal [129]. As a result, CAR γδ T cell tumour trafficking was enhanced in a non-small cell lung cancer mouse model, facilitating disease control.

### 4.7. CAR Alternatives

An elegant CAR alternative known as a T cell receptor fusion construct (TruC) comprises a fusion of a synthetic antigen recognition domain directly to CD3ε, enabling the complex to assimilate within the TCR/CD3 complex. Juraske et al. designed a CD19-specific TRuC construct that utilises the unique γδ TCR architecture to leverage both innate and adaptive immune activity [100]. Through these means, dual tumour-targeting specificity was achieved via the endogenous γδ TCR and the CD19-specific scFv.

Kitidee et al. described another strategy whereby Vδ2 cells were engineered to express a T cell engager specific for tumour-associated GD2 and CD3, providing yet another dual targeting approach [130].

Building on this, Vδ2 cells have also been engineered to co-express a CAR (targeted against HLA-G) together with a T cell engager specific for PD-L1 and CD3, thereby conferring multiple routes of tumour attack and with confirmed efficacy in vivo [131].

### 4.8. Chemotherapy-Resistant Vδ2 Cells

Lamb et al. have engineered ex-vivo-expanded Vδ2 cells to over-express O(6)-methylguanine DNA methyltransferase (MGMT), thereby conferring resistance to temozolomide [132]. This alkylating agent is used as standard of care in the management of glioblastoma and also upregulates NKG2D ligands in tumours. The efficacy of combination therapy using temozolomide and MGMT-engineered Vδ2 cells was demonstrated in orthotopic PDX models of glioblastoma [132].

## 5. Clinical Experience of Engineered γδ T Cell Therapy in Cancer

Adoptive immunotherapy using unmodified γδ T cells has been practised over many years. While this approach has proven safe even in the allogeneic setting, its efficacy has been modest. The largest study was recently reported by Xu et al., in which 414 infusions of allogeneic δ2 cells were administered to 132 patients with lung and liver cancers [133].

By contrast, the clinical experience with gene-modified γδ T cell therapy remains at an early stage. The majority of registered studies are phase I, reflecting ongoing efforts to optimise γδ-specific engineering, expansion and manufacturing strategies (Table 1). Moreover, most employ an allogeneic approach. This underscores the principal advantages of γδ T cells over conventional αβ T cells; namely, the profoundly reduced risk of GvHD, suitability for off-the-shelf manufacturing and growing clinical interest.

Clinical data from these studies are limited and may be summarised as follows. A collaborative study between the University of Alabama at Birmingham and IN8Bio evaluated MGMT-engineered Vδ2 T cell immunotherapy of glioblastoma in phase I [134] and phase II trials [135]. Cells were repeatedly infused into the surgical resection cavity. Fifteen patients were recruited, of whom 8 were treated safely without dose-limiting toxicity. However, the phase II study was terminated in 2024 (https://www.fiercebiotech.com/biotech/in8bio-halting-phase-2-glioblastoma-trial-and-laying-nearly-half-workforce-focus-leukemia, accessed on 29 January 2026).

**Table 1:** Clinical trials of genetically engineered γδ T cell therapies registered up to January 2026 ^1^.

Year Opened	Trial ID	Status	Phase	Target Antigen	Indication	γδ Subset	Source	Reference
2017	NCT02656147	Unknown	1	CD19	R/R CD19^+^ malignancies	Unspec. ^2^	Allo	N/A
2019	NCT03885076	Unknown	Ob.	Unspec.	AML	Unspec.	Auto	N/A
2019	NCT04107142	Unknown	1	NKG2DL	R/R solid tumours	Unspec.	Allo	N/A
2020	NCT04165941	Active, not recruiting	1	N/A	Glioblastoma	Vδ2	Auto	[134]
2020	NCT04702841	Unknown	1	CD7	CD7^+^ T cell malignancy	Unspec.	Unspec.	N/A
2021	NCT04735471	Terminated (commercial decision, pivot to autoimmunity)	1	CD20	B cell Malignancies	Unspec.	Allo	[136]
2022	NCT05554939	Recruiting	1/2	CD19	R/R B-cell NHL	Unspec.	Allo	N/A
2022	NCT05388305	Unknown	N/A	Unspec.	R/R post-transplant AML	Unspec.	Unspec.	N/A
2023	NCT05664243	Active, not recruiting	2	N/A	Glioblastoma	Vδ2	Auto/Allo	[135]
2023	NCT06372236	Completed	1	B7-H3	Advanced solid tumours	Vδ1	Allo	N/A
2023	NCT06056752	Recruiting	1	CD19	CD19^+^ B-ALL	Unspec.	Allo	N/A
2023	NCT06092047	Recruiting	1	CD19	CD19^+^ B cell malignancy	Unspec.	Allo	N/A
2024	NCT05302037	Recruiting	1	NKG2DL	Advanced malignancies	Unspec.	Allo	[137]
2024	NCT06018363	Recruiting	1/2	B7-H3	Malignant brain glioma	Unspec.	Allo	[138]
2024	NCT06150885	Recruiting	1/2	HLA-G ^3^	R/R NSCLC, TNBC, CRC, GBM	Vδ2	Allo	N/A
2024	NCT06193486	Recruiting	1	PSCA	mCRPC	γδ-enriched	Auto	[139]
2024	NCT06480565	Active, not recruiting	1/2	CD70	Clear Cell Renal Cell Carcinoma	Vδ1	Allo	[140]
2024	NCT06592092	Recruiting	N/A	B7H3	Meningeal metastases from B7H3^+^ solid tumours	Unspec.	Allo	[141]
2025	NCT06838832	Recruiting	1/2	CD19	R/R B-NHL	Unspec.	Allo	N/A
N/A	NCT07120607	Not yet recruiting	1	CD7	R/R CD7^+^ leukaemia/lymphoma	Unspec.	Allo	N/A

^1^ https://clinicaltrials.gov/, accessed on 26 January 2026. ^2^ Abbreviations: Allo—allogeneic; AML—acute myeloid leukaemia; Auto—autologous; B-ALL—B cell acute lymphoblastic leukaemia; B-NHL—B cell non-Hodgkin’s lymphoma; CRC—colorectal cancer; GBM—glioblastoma multiforme; mCRPC—metastatic castrate-resistant prostate cancer; N/A—not available; NKG2DL—ligands of the NKG2D receptor; NSCLC—non-small cell lung cancer; Ob.—observational; PSCA—prostate stem cell antigen; R/R—relapsed/refractory; TNBC—triple-negative breast cancer; Unspec—unspecified. ^3^ CAR-BiTE (bispecific T cell engager).

Adicet Bio evaluated ADI-001, an allogeneic CD20 CAR Vδ1 cell therapy, in patients with B cell malignancies (GLEAN-1 clinical trial; NCT04735471). Early data were very encouraging, with a complete response seen in 4 of 6 treated patients in the absence of either GvHD or dose-limiting toxicity [136]. However, responses did not appear to be sufficiently durable for further development and the trial was halted prematurely followed by a pivot in development of this asset for autoimmune disease (https://www.oncologypipeline.com/apexonco/adicets-cancer-u-turn, accessed on 26 January 2026).

CytoMed are evaluating allogeneic NKG2DL-targeted CAR γδ T cells in a basket study comprising a range of malignancies (ANGELICA trial, NCT05302037) [137]. Transient CAR expression is achieved by mRNA electroporation and patients receive ZOL and IL-2, suggesting that Vδ2 cells are present in the product.

Unicet Biotech are undertaking a phase I clinical trial, in which B7-H3 CAR γδ T cells are administered monthly using the intrathecal route in patients with recurrent glioblastoma [138]. Seven patients have been treated in this dose escalation study without dose-limiting toxicities or GvHD noted to date. Three of seven subjects had a partial response and disease control was seen in all cases.

The Cancer Institute and Hospital of the Chinese Academy of Medical Sciences is evaluating allogeneic B7-H3 CAR γδ T cells administered as a single intrathecal dose to patients with leptomeningeal metastases that derive from tumours expressing this target [141]. Thus far, two subjects with lung adenocarcinoma have been treated, achieving stable disease in both cases. One treatment-related absence seizure was also reported.

## 6. Lessons Learned from Clinical Experience to Date

Thus far, immunotherapy using engineered γδ T cells has achieved excellent safety. Moreover, a promising early efficacy signal was observed with ADI-001, represented by the attainment of complete responses in 4 out of 6 lymphoma patients. These data demonstrate the clear potential of allogeneic Vδ1 CAR-T cells. Nevertheless, the absence of sustained responses and the subsequent discontinuation of this oncology program underscore substantial hurdles in maintaining long-term cell persistence of these allogeneic cells, emphasising the need for enhanced armouring or priming approaches or improved systems to enable these cells to evade host cell rejection. The strategic shift in ADI-001 development toward autoimmune indications is consistent with clinical data generated in the autologous αβ T cell setting, which suggest that requirements for T cell persistence may be reduced in that setting [142].

Although the phase I trial of MGMT-engineered Vδ2 cells by IN8Bio established a favourable safety profile, the discontinuation of the phase II study highlights the formidable challenges associated with treating solid tumours such as glioblastoma, even when employing a chemotherapy-resistant cell product. This outcome reinforces the need for combinatorial strategies capable of simultaneously addressing the profoundly immunosuppressive tumour microenvironment characteristic of this tumour type. Moreover, as indicated in the preceding section, CAR engineering directed against B7-H3 has achieved some early signs of success when γδ T cells have been administered using the intrathecal route in this and other indications. However, the durability of these responses remains unclear at this time.

## 7. Future Directions and Outstanding Questions

γδ T cells sit within a population known as unconventional T cells with several shared properties. These include non-classical antigen recognition mechanisms, potential for rapid (innate-like) responsiveness, distinctive tissue tropism attributes, markedly reduced potential for GvHD, amenability to advanced genetic engineering strategies and consequent suitability for off-the shelf manufacture as cell therapy products [143,144].

Several challenges remain to be addressed before the full therapeutic potential of engineered γδ T cells can be realised. A deeper understanding of the biological differences between γδ T cell subsets will be critical to identify specific strategies for the effective use of these products. A key outstanding question is which γδ T cell subset should be selected to maximise efficacy. The distinct tissue distribution of δ2 and non-δ2 γδ T cells may have implications for their preferred application in specific disease types. Conceptually, the mainly circulatory distribution of δ2 cells renders them of potentially greatest utility in the treatment of haematological malignancies such as leukaemias. By contrast, the tissue-resident nature of non-δ2 cells may better position them for the treatment of solid tumours that originate from or metastasise to parenchymal organs.

Hybrid approaches may also prove valuable in this regard. For example, Vδ2 cells expanded in TGF-β not only acquire enhanced anti-tumour activity and fitness but also exhibit alterations in chemokine receptor expression (e.g., CXCR4 upregulation) and markers of tissue residency (e.g., CD103 expression) that may favour application against solid tumours. Alternatively, products that contain a mixture of γδ T cell subtypes may be a better option for some tumour types. In parallel, exploring expansion methods to enrich beneficial γδ T cell subsets while enhancing fitness and cytotoxic efficacy and limiting AICD and other unfavourable attributes (e.g., IL-17 production) will be necessary to drive clinical translation. Manufacturing processes must be designed while conscious of clinical translation GMP regulations and maximising the use of automation, closed processing, scalability and digital data storage.

Chimeric antigen receptor designs should be tailored for greater compatibility with the γδ T cell chassis. Conventional CAR designs used in αβ T cells promote tonic-signalling-induced exhaustion and fail to harness unique γδ T cell functions, such as innate stress sensing and ADCC. For application in the treatment of solid tumours, questions remain as to whether CAR-engineered γδ T cells are preferred or instead whether T cell engagers, native TCR exploitation, TruC, bispecific-engager-based targeting or use of CCRs or recently described opsonin-secreting strategies may achieve more impressive clinical impact. Other signalling modules that operate in native γδ T cell biology may also warrant exploration in this context. In particular, high-throughput screening of CAR libraries may facilitate the identification of optimised modular receptor systems that enable durable γδ T cell anti-tumour function [145].

Enhanced persistence accompanied by reduced exhaustion remains another key challenge associated with the use of these cells. Already we have seen encouraging data generated via cellular armouring with IL-15, IL-18 or dnTGFβRII and with PD-1 blockade. However, the field lacks sufficient direct comparison of these strategies and there is also a need to rationalise the investigation of derived combinatorial approaches.

Pharmacokinetics and pharmacodynamics of infused allogeneic γδ T cells also warrant further consideration. Cytokine armouring systems can enable improved expansion and persistence of these cells while chemokine receptor engineering can direct the migration of these cells into appropriate tissues, depending on tumour type. The allogeneic nature of these cells by definition means that they may be susceptible to host rejection. However, veto systems may assist to delay rejection, such as CARs targeted against CD70 [140] or 4-1BB ligand, combined with CD58 knockout [146].

## 8. Conclusions

Genetically engineered γδ T cells represent a promising advance with the potential to provide a scalable allogeneic off-the-shelf therapy for a variety of tumour types. The safety of these cells has been excellent with reduced toxicity due to cytokine release compared to αβ T-cells. Moreover, there is little clinical evidence that γδ T cells can elicit GvHD, obviating the need for complex genome editing approaches that are required for the allogeneic deployment of engineered αβ T cells. Early clinical trial data are limited but promising and continued clinical evaluations of CAR and non-CAR γδ T cell platforms to define their persistence, safety and efficacy will be essential to guide future applications of γδ T cell therapeutics.

## Data Availability

No new data were created in this review.

## Abbreviations

The following abbreviations are used in this manuscript:

aAPC	Artificial antigen-presenting cell
AAV	Adeno-associated virus
ADCC	Antibody-dependent cellular cytotoxicity
AICD	Activation-induced cell death
Allo	Allogeneic
AML	Acute myeloid leukaemia
AMPK	AMP-activated protein kinase
Auto	Autologous
BrHPP	Bromohydrin pyrophosphate
BTN	Butyrophilin
CAR	Chimeric antigen receptor
CCR	Chimeric co-stimulatory domain
CISH	Cytokine-inducible SH2 containing protein
CRC	Colorectal carcinoma
CRISPR	Clustered regularly interspaced short palindromic repeats
DC	Dendritic cell
dnTGFβRII	Dominant-negative TGF-β receptor II
DNAM-1	DNAX accessory molecule-1
DOT	Delta one T cell
FDA	U.S. Food and Drug Administration
GBM	Glioblastoma
GMP	Good Manufacturing Practice
G-Rex^®^	Gas-permeable rapid expansion
GvHD	Graft versus host disease
HIF	Hypoxia-inducible factor
IFN	Interferon
IL	Interleukin
MACS^®^	Magnetic cell separation
Mb	Membrane bound
mCRPC	Metastatic castrate-resistant prostate carcinoma
MDSC	Myeloid-derived suppressor cells
MGMT	O(6)-methylguanine DNA methyltransferase
MHC	Major histocompatibility complex
N/A	Not available
N-BP	Aminobisphosphonate
NFAT	Nuclear factor of activated T cells
NHL	Non-Hodgkin lymphoma
NK	Natural killer
NKG2DL	Natural killer group 2 D receptor ligands
NSCLC	Non-small cell lung carcinoma
Ob.	Observational
pAg	Phosphoantigen
PBMC	Peripheral blood mononuclear cell
PD-L1	Programmed death ligand 1 (PD-L1)
PSCA	Prostate stem cell antigen
R/R	Relapsed/refractory
scFv	Single-chain variable fragment
TCR	T cell receptor
TGF-β	Transforming growth factor β
TME	Tumour microenvironment
TNBC	Triple-negative breast carcinoma
TNF-α	Tumour necrosis factor α
TRAIL	TNF-related apoptosis-inducing ligand
TRuC	T cell receptor fusion construct
TRUCK	T cells redirected for universal cytokine-mediated killing
Unspec.	Unspecified
ZOL	Zolendronic acid
